# Thermal Decomposition of Brominated Butyl Rubber

**DOI:** 10.3390/ma14226767

**Published:** 2021-11-10

**Authors:** Wei Zhang, Yang Zang, Yanli Lu, Weisheng Lin, Shengyun Zhao, Jinping Xiong

**Affiliations:** 1 School of Materials Science and Engineering, Beijing University of Chemical Technology, Beijing 100029, China; 2019200387@mail.buct.edu.cn (W.Z.); 2019310030@mail.buct.edu.cn (Y.Z.); 2019310035@mail.buct.edu.cn (Y.L.); 2 School of Ecology and Resource Engineering, Wuyi University, Mount Wuyi, Nanping 354300, China; 17601537369@163.com (W.L.); Zhaosy@126.com (S.Z.); 3 Fujian Provincial Key Laboratory of Ecological Industry Green Technology, Mount Wuyi, Nanping 354300, China

**Keywords:** brominated butyl rubber, thermal decomposition, lifetime

## Abstract

The thermal decomposition of brominated butyl rubber under air atmosphere was investigated by thermogravimetry (TG) and derivative thermogravimetry (DTG) at various heating rates. The kinetic parameters were evaluated by TG and the isoconversional method developed by Ozawa. One prominent decomposition stage was observed in the DTG curves at high heating rates, while an additional small peak was observed at low heating rates. The apparent activation energy determined using the TG method ranged from 219.31 to 228.13 kJ·mol^−1^ at various heating rates. The non-isothermal degradation was found to be a first-order reaction, and the activation energy, as determined by the isoconversional method, increased with an increase in mass loss. The kinetic data suggest that brominated butyl rubber has excellent thermal stability. This study can indirectly aid in improving rubber pyrolysis methods and in enhancing the heat resistance of materials.

## 1. Introduction

Brominated butyl rubber (BIIR) is more advantageous than ordinary butyl rubber and is finding increasing applications in many fields [1,2]. Research on the thermal oxidative degradation of BIIR has gained significant attention. This is because BIIR is prone to degradation during long-term use in heat-resistant products, and the study of its performance degradation upon thermal decomposition can aid in developing methods to improve its thermal aging properties. Additionally, pyrolysis, which is commonly employed in rubber recycling industries, can be effectively utilized to recover resources and energy, thus promoting the progress of waste plastic processing [3]. In this regard, a study on the thermal decomposition kinetics of rubber is an effective approach.

There have been a considerable number of studies on the thermal degradation of rubber materials [4,5,6,7] and other polymeric materials [8,9,10] using different calculation methods and model fitting to experimental data. Liu et al. [4] studied the effect of vulcanization on the pyrolysis of natural rubber, butadiene rubber, and styrene–butadiene rubber. Based on the activation energy distribution obtained using the distributed activation energy model and the subsequent verification by a model-free method, they suggested that the decomposition of rubber occurs predominantly via a chain reaction. Hu et al. [5] studied the co-pyrolysis of four kinds of real-world plastics and tires in a thermogravimetry–Fourier-transform infrared instrument, and kinetic analysis using a single first-order reaction model indicated that the co-pyrolysis occurred in three stages. The DTG curves revealed that the pyrolysis of tire proceeded in two steps, while the pyrolysis of plastics proceeded in a single step. The detailed analysis of the degradation kinetics of an EPDM–NBR blend by Basha et al. [6] provided insights into the degradation mechanism. Rasam et al. [7] investigated the kinetics and thermal behavior of Spirulina microalgae, sugarcane bagasse, scrap tire, and their mixtures from the TG and DTG curves. According to the kinetic data, they estimated the relationship among the thermodynamic parameters (ΔG, ΔH, and ΔS) using TG. Das et al. [8] and Xu et al. [9] studied the thermal decomposition of plastics in the linear heating mode or at a high heating rate by dynamic thermogravimetry. They used model-free methods such as the Ozawa–Flynn–Wall (OFW) method, Kissinger–Akahira–Sunose (KAS) method, and Friedman method for calculating the activation energy. The reaction kinetics were predicted using model fitting methods (including Coats–Redfern and Criado methods). Using isoconversional methods, Das et al. determined the activation energy distribution at each stage of degradation and its influence on the degradation process. Wang et al. [10] investigated the thermal degradation kinetics and non-isothermal crystallization kinetics of mEBHC/HF–PP to predict the thermal stability on the basis of the activation energy obtained using TG analysis and DTG.

The thermal degradation kinetics of BIIR have not been reported yet. In this study, the thermal stability of BIIR was investigated by TG and DTG under air atmosphere. TG was performed at different heating rates to study the impact of heating rate on the thermal decomposition of BIIR. To quantitatively study the thermal stability of BIIR, the reaction pathway was obtained from the Crane equation using the kinetic parameters of thermal decomposition, such as the activation energy of decomposition and frequency factor. This kinetic analysis provides insights into the mechanism of pyrolysis of BIIR, which can subsequently help in improving rubber pyrolysis methods and in enhancing the heat resistance of materials.

## 2. Materials and Methods

### 2.1. Raw Materials

**Figure 1:**
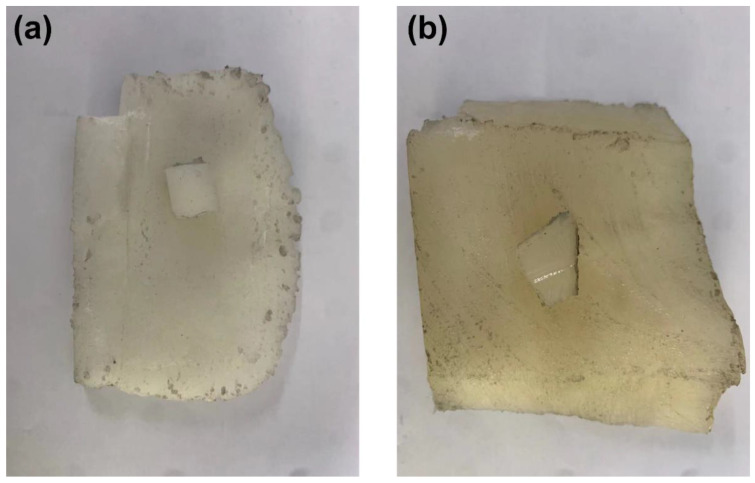
The raw rubber material (**a**) and the sample (**b**).

BIIR2222 was manufactured by Exxonmobil (Irving, TEXAS, USA). The compounding ingredients (phr) were BIIR (100), carbon black N660 (55), sulfur (1), paraffin oil (1), stearate (1.5), zinc oxide (6), and magnesium oxide (2). An image of the raw rubber material is shown in Figure 1a, and an image of the sample is shown in Figure 1b.

### 2.2. Specimen Preparation

In an open mill (X (S) K-160 open mill, Shanghai No. 1 Rubber Machinery Factory) at room temperature, BIIR was added, rolled to 2 mm thick, and a thin film was passed out several times. A vulcameter (P3555C2 vulcameter, Beijing, Central Peak Chemical Machinery experimental works) was used to measure the curing time. Curing was performed for 12 min in a platen press (25 t platen press, Zhejiang Orient Machinery Co., Ltd., Huzhou, China) at a curing temperature of 160 °C to prepare 2 mm thick specimens of BIIR.

### 2.3. TG Analysis

TG analysis of BIIR in the STARe system thermal gravimetric analyzer (Switzerland METTLER-TOLEDO’s product, Zürich, Switzerland) was performed under air atmosphere at an air flow rate at 20 mL/min and heating rates of 0.5 °C/min, 1 °C/min, 2 °C/min, 5 °C/min, 10 °C/min, and 20 °C/min in the temperature range 30–420 °C.

## 3. Result and Discussion

### 3.1. Thermal Decomposition

**Figure 2:**
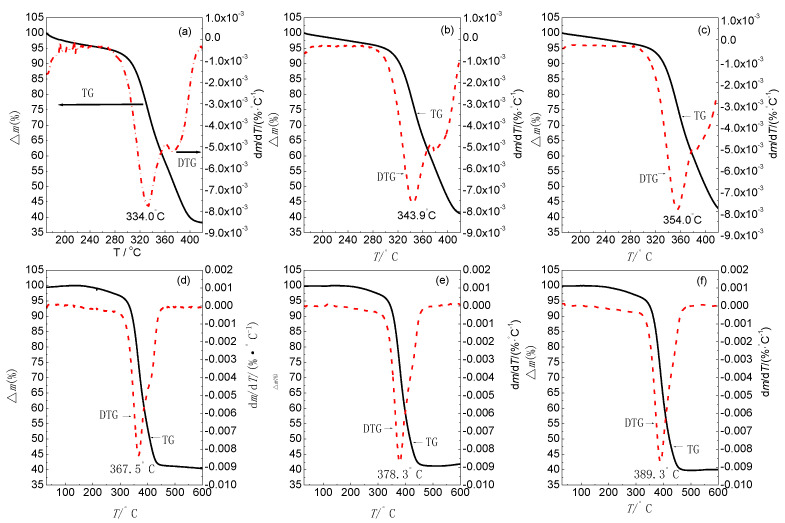
TG and DTG curves of BIIR in air at different heating rates: (**a**) 0.5 °C min^−1^, (**b**)1 °C min^−1^, (**c**) 2 °C min^−1^, (**d**) 5 °C min^−1^, (**e**) °C min^−1^, and (**f**) 20 °C min^−1^.

Figure 2 shows the TG–DTG curves obtained at different heating rates. It is evident that there was a rapid weight loss in the temperature range 334.0–389.3 °C at all heating rates. This is consistent with the reported melting temperature of BIIR (386 °C) [11]. The weight loss peaks shifted to higher temperature with increasing heating rate (Figure 1 and Table 1). Table 1 shows that the decomposition temperature (*T_pi_*) of BIIR lay between 334.0 °C (at 0.5 °C·min^−1^) and 389.3 °C (at 20 °C·min^−1^), while the weight loss was between 25.5% and 28.0%. The molecular chains of the specimen gradually underwent scission with increased heating rate during the thermal analysis. The weight loss peak shifted to a higher temperature as the relaxation time of the molecular chain movement could not keep up with experimental observation time. Because the molecular chain movement and the activation energy of decomposition have an indirect relationship with the molecular chain relaxation and temperature, DTG can be employed to analyze the decomposition kinetics of BIIR. A careful inspection of the DTG curves in Figure 1 shows the appearance of an additional peak, corresponding to rapid weight loss, at low heating rates (0.5 °C min^−1^, 1 °C·min^−1^, and 2 °C·min^−1^). However, the peak size decreased and the peak ultimately disappeared with increasing heating rate. This is because rapid heating promotes pyrolysis at high temperatures, whereby the reactions at a higher temperature are delayed. However, the reaction steps can be precisely identified at a low heating rate [12].

BIIR is prepared by reacting butyl rubber and a small amount of bromine at a certain temperature, and its structure is as follows [13]:

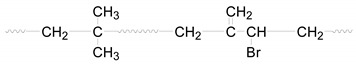



During its thermal decomposition, the –C–Br bond dissociates first owing to low bond energy (Table 2), consequently resulting in the formation of hydrogen bromide at ~230 °C [14]. However, no significant changes can be observed in the TG/DTG curves (Figure 1) due to the low bromine content in the compound. Rapid weight loss is observed from 330 to 390 °C primarily due to the dissociation of the –C–C– bonds in the main chain. The formation of oligomers upon chain scission is accompanied by the generation of a large amount of hydrocarbon gas in the presence of oxygen, and a sharp weight loss is observed at a lower heating rate in the temperature range 370–380 °C. This weight loss is much smaller than that arising from the dissociation of the main chain [15]. The thermal decomposition pathway can be represented as follows:

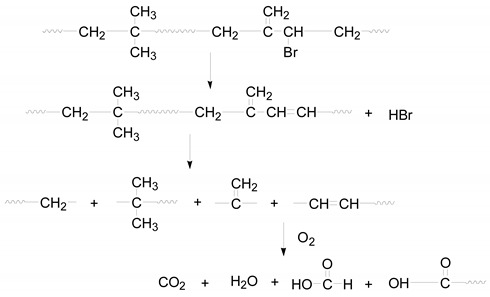



**Table 2:** Bond energies in BIIR.

Bond	–C=C–	–C–H	–C–C–	–C–Br
Bond energy (KJ/mol)	605	420	346	292

### 3.2. Activation Energy and Reaction Order of Thermal Decomposition

#### 3.2.1. TG Method

The relationship between the extent of weight loss (α) and the time of thermal decomposition can be explained by Equation (1) as follows [16]:

(1) $$\frac{d\alpha }{dt}=A{\left(1-\alpha \right)}^{n}e^{-E/RT},$$

where *A* represents the preexponential factor, *E* is the activation energy, *T* is the absolute temperature, *R* is the gas constant, and *n* is the reaction order. Under non-isothermal conditions, *A* in Equation (1) is related to the temperature as *A* = *A*_0_*T*^1/2^. Using this relation, Equation (1) can be transformed into Equation (2) as follows:

(2) $$\frac{d\alpha }{dt}=\frac{A_{0}}{\beta }T^{1/2}e^{-E/RT}{\left(1-\alpha \right)}^{n},$$

where *β* = d*T*/d*t*.

An approximated integrated form, Equation (3), can be derived after inserting Equation (2) according to the Doyle method [17]. The values of *E* and *A* can be calculated using the TG method.

(3) $$\mathrm{lg}\left[\mathrm{lg}{\left(1-a^{1/2}\right)}^{-1}\right]=\mathrm{lg}\frac{AE}{2.3R\beta }-2.315-0.457\frac{E}{RT}.$$

**Figure 3:**
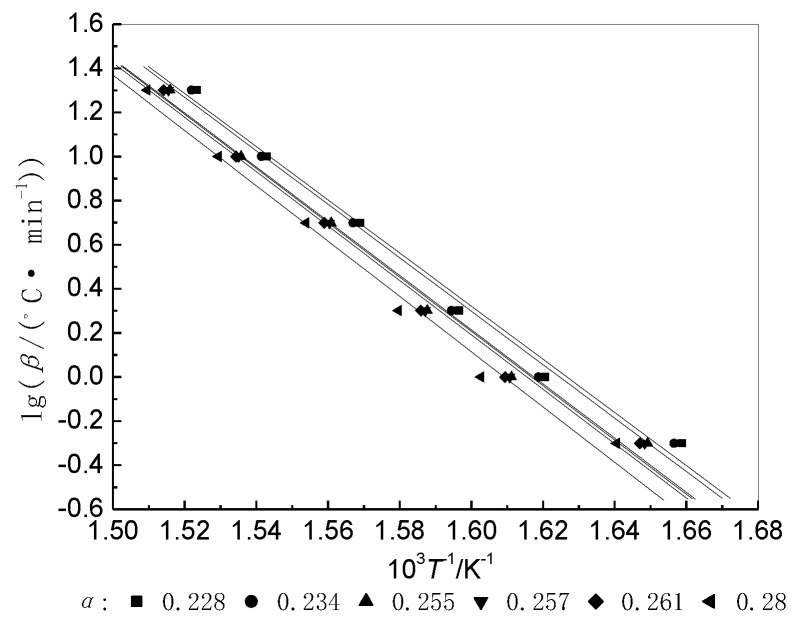
Ozawa plots (based on the TG method) for the activation energy of decomposition of BIIR at different α values.

An Ozawa plot of lg*β* against 1/*T* for different values of *α* yields a set of parallel straight lines (Figure 3). The values of *E* and *A* (Table 3) can be obtained respectively from the slopes and intercepts of the straight lines by linear regression, with regression coefficient *r*. The average correlation coefficient *r* obtained by the TG method is greater than 0.99 (close to 1); accordingly, the activation energy calculated using this method is more accurate [18].

The order of thermal decomposition (*n*) of BIIR can be obtained from the Crane equation, as shown in Equation (4)):

(4) $$\frac{d\ln\beta }{d(1/T_{pi})}=-\frac{E}{nR}-2T_{pi}.$$

A plot of ln *β* against 1/*T_pi_* yields a straight line. The reaction order *n* (Table 3) can be obtained by substituting *E* and *T_pi_* in Equation (4). The calculated values of *n* were close to 1, suggesting that the thermal decomposition of BIIR is a first-order reaction.

#### 3.2.2. Isoconversional Procedure (Integral Method)

To determine the activation energy *E* of BIIR decomposition more accurately, an isoconversional method developed by Ozawa [19] was used. The values of *E* and *A* were obtained using Equation (5) as follows:

(5) $$\mathrm{lg}\beta =\mathrm{lg}\frac{AE}{-R\ln(1-\alpha )}-2.315-0.457\frac{E}{RT}.$$

**Figure 4:**
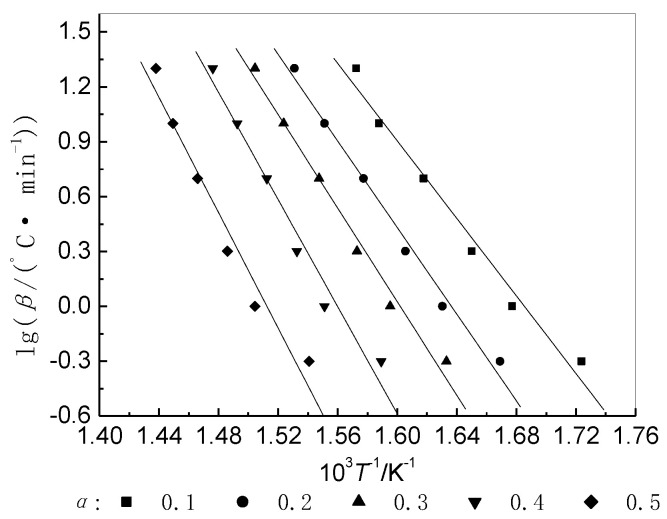
Isoconversional plots of BIIR decomposition in air.

It is evident that the values of *E* and *A* can be obtained from the slope and intercept of the straight line of lg*β* against 1/*T* (Figure 4) at a particular value of (1 − *α*) (Table 3).

**Figure 5:**
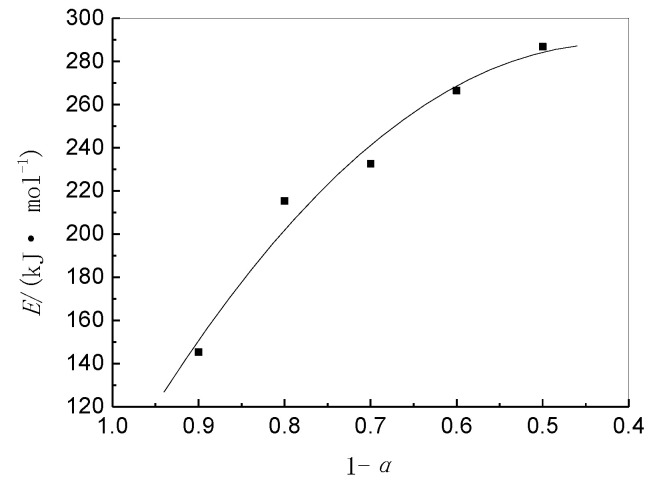
Plot of apparent activation energy (*E*) for BIIR decomposition in air as a function of *α*.

Table 4 shows that the activation energy for the thermal decomposition of BIIR is high and is dependent on *E*, *A*, and *α*. With an increase in the extent of weight loss, the *E* values tend to increase gradually (Figure 5). It is, thus, concluded that dissociation of the main chain, which results in weight loss, requires a higher amount of energy.

### 3.3. Lifetime of Thermal Decomposition

The life of polymeric materials is affected by the thermal decomposition during thermal aging, even at high temperatures. Thermal aging is also governed by the reaction kinetics, and Equation (6) given by Dakin [20] can be used to predict the lifetime at any temperature.

(6) $$\mathrm{lg}\tau =\alpha \frac{1}{T}+b,$$

where *τ* is the lifetime at temperature *T*, and *α* and *b* are constants derived by integrating Equation (7), which represents the dissociation

(7) $$-\frac{d\alpha }{dt}=A\alpha^{n}E^{-E/RT}.$$

Variable separation yields Equation (8).

(8) $$\int_{1}^{\alpha }-d\alpha /\alpha^{n}=Ae^{-E/RT}\int_{0}^{\tau }dt.$$

When *n* = 1,

(9) $$\mathrm{lg}\tau =E/2.303RT+\mathrm{lg}\left[(\ln\alpha_{\tau })/A\right].$$

*α* and *α_τ_* are respectively the remaining percentage and the residual percentage at the end of life. Comparing Equations (6) and (9), we get

$$\alpha =E/2.303R,b=\mathrm{lg}(-A^{-1}\ln\alpha_{\tau }).$$

**Figure 6:**
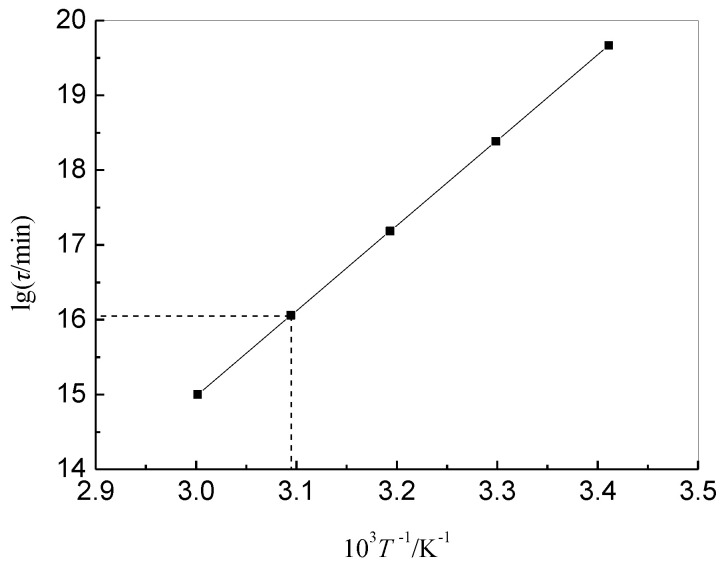
Prediction of estimated lifetime for *α_τ_* = 95%.

Taking the minimum *E* for 219.31 kJ·mol^−1^ and the maximum *A* for 8.55 × 10^17^ min^−1^ (ln*A* = 41.29) in Table 3, when defining a weight loss of 5% to be the index of the end of life for the brominated butyl rubber, it was calculated that *α* = 1.14 × 10^4^, *b* = −19.22. Figure 6 shows the life curve, lg*τ* against 1/*T* of BIIR at 5% weight loss. The lifetime *τ* at a particular temperature and the limit of temperature *T* at an arbitrary lifetime can be estimated. It is evident that the lifetime could extend to more than 50 years at 30 °C in air.

## 4. Conclusions

The thermal decomposition of BIIR in air proceeds in three steps. The first step is the formation of hydrogen bromide upon the dissociation of the C–Br bond, the second step is the dissociation of the main chain, and the third step is the generation of hydrocarbon gases through oxidation. The activation energy of thermal decomposition at various heating rates, as calculated by the TG method, ranged from 219.31 to 228.13 kJ·mol^−1^, and the reaction order of thermal decomposition was 1. Analysis using the isoconversional method developed by Ozawa revealed that the activation energy increased with an increase in mass loss. The lifetime estimated from the relationship between the activation energy and weight loss suggested that the lifetime of BIIR was more than 50 years at 30 °C for a weight loss of 5%. The findings of this study can indirectly help in enhancing the heat resistance of materials.

## Figures and Tables

**Table 1:** Decomposition temperatures (T_pi_/°C) of BIIR at different heating rates in air.

*β* (°C·min^−1^)	Δm (%)	T_pi_ (°C)
0.5	73.9	334.0
1	77.2	343.9
2	76.6	354.0
5	74.5	367.5
10	74.3	378.3
20	72.0	389.3

**Table 3:** Kinetic parameters of BIIR decomposition.

*β* (°C min^−1^)	TG Method			
	*E* (kJ·mol^−1^)	*R*	ln*A* (min^−1^)	*n*
0.5	224.41	−0.9946	40.67	1.05
1	219.31	−0.9949	39.87	1.03
2	220.55	−0.9946	40.08	1.04
5	223.64	−0.9943	40.56	1.05
10	224.01	−0.9945	40.62	1.05
20	228.13	−0.9940	41.29	1.07

**Table 4:** Apparent activation energy (*E*) and frequency factor (*A*) at different fractional mass loss (*α*) values for BIIR decomposition in air.

1 − *α*	0.9	0.8	0.7	0.6	0.5
*E* (kJ·mol^−1^)	145.32	215.33	232.66	266.50	286.75
ln*A* (min^−1^)	34.43	38.27	41.24	46.90	49.41

## Data Availability

No new data were created or analyzed in this study. Data sharing is not applicable to this article.
